# A Simple Assay for Ultrasensitive Colorimetric Detection of Ag^+^ at Picomolar Levels Using Platinum Nanoparticles

**DOI:** 10.3390/s17112521

**Published:** 2017-11-02

**Authors:** Yi-Wei Wang, Meili Wang, Lixing Wang, Hui Xu, Shurong Tang, Huang-Hao Yang, Lan Zhang, Hongbo Song

**Affiliations:** 1 Key Laboratory of Predictive Microbiology and Chemical Residual Analysis, College of Food Science, Fujian Agriculture and Forestry University, Fuzhou 350002, China; wangyw@fafu.edu.cn (Y.-W.W.); fj150823@163.com (L.W.); xhuifst@163.com (H.X.); 2 The Key Lab of Analysis and Detection Technology for Food Safety of the MOE, State Key Laboratory of Photocatalysis on Energy and Environment, College of Chemistry, Fuzhou University, Fuzhou 350108, China; wang641132431@163.com (M.W.); hhyang@fzu.edu.cn (H.-H.Y.); 3 Department of Pharmaceutical Analysis, Faculty of Pharmacy, Fujian Medical University, Fuzhou 350108, China

**Keywords:** platinum nanoparticles, peroxidase-mimic activity, colorimetric sensor, silver ions detection

## Abstract

In this work, uniformly-dispersed platinum nanoparticles (PtNPs) were synthesized by a simple chemical reduction method, in which citric acid and sodium borohydride acted as a stabilizer and reducer, respectively. An ultrasensitive colorimetric sensor for the facile and rapid detection of Ag^+^ ions was constructed based on the peroxidase mimetic activities of the obtained PtNPs, which can catalyze the oxidation of 3,3’,5,5’-tetramethylbenzidine (TMB) by H_2_O_2_ to produce colored products. The introduced Ag^+^ would be reduced to Ag^0^ by the capped citric acid, and the deposition of Ag^0^ on the PtNPs surface, can effectively inhibit the peroxidase-mimetic activity of PtNPs. Through measuring the maximum absorption signal of oxidized TMB at 652 nm, ultra-low detection limits (7.8 pM) of Ag^+^ can be reached. In addition to such high sensitivity, the colorimetric assay also displays excellent selectivity for other ions of interest and shows great potential for the detection of Ag^+^ in real water samples.

## 1. Introduction

Peroxidase is a hemin-containing oxidase that can catalyze the chemical reactions in a variety of biological processes by binding electrons to specific substrates. Since the peroxidase is capable of catalyzing the formation of colored products in very low concentrations, it has become the most frequently used enzyme in enzyme-linked immunosorbent assay and widely used in the detection of various substances through the combination with other enzymes to form multi-enzyme systems [1]. However, the inherent defects of natural enzymes, such as limited source, low stability, complex purification processes, and expensive purification costs, restricted their production and application. Therefore, great efforts have been made to synthesize mimetic enzymes. In virtue of chemical reactions that happen mainly on the surface of nanozymes, different surface modification methods are studied to improve catalytic activity, substrate specificity, and stability [2]. Since the first discovery of Fe_3_O_4_ nanoparticles [3], many inorganic nanomaterials with enzyme-mimic activities are explored and widely used in biomedical and environmental monitoring, such as glutathione-capped palladium or platinum nanoparticles [4,5], AuPt nanoparticles [6], gold nanoparticles@carbon shells [7], cobalt oxyhydroxide nanoflakes [8], g-C_3_N_4_/Pt nanoparticles [9], and MoS_2_ nanosheets [10].

Silver ions (Ag^+^), as one of the heavy metal ions, is highly toxic to bacteria, viruses, algae, and fungi. Due to the unique antibacterial properties, Ag^+^ has been widely used in cosmetics, building materials and medical products [11,12]. The excessive uptake of Ag^+^ may lead to many serious diseases, including cytotoxicity, organ failure, and mitochondrial dysfunction [13]. Due to the hazardous effects of Ag^+^, the maximum allowable level of Ag^+^ in drinking water is limited by the U.S. Environmental Protection Agency (EPA) to about 900 nM [14]. The U.S. EPA reported that the concentration of Ag^+^ higher than 1.6 nM is toxic to fish and micro-organisms [15]. Hence, it has become increasingly important to develop a simple method for the sensitive detection of Ag^+^ in the environment and biological samples.

Over the past decades, many analytical methods have been developed to detect Ag^+^ with high sensitivity and selectivity, involving inductively-coupled plasma mass spectrometry (ICP-MS) [16], atomic absorption spectroscopy (AAS) [17], and atomic emission spectrometry (AMS) [18]. The requirements of large instruments, highly-trained operators, and lengthy sample preparation procedures in these methods, impede their capacity to routine and in situ detection. In contrast, chemical sensors provide an excellent platform to make up for the deficiency [19]. A novel silver-specific RNA-cleaving DNAzyme has been selected in vitro for sensitive fluorescence detection of Ag^+^ [20]. Colorimetric sensors offer great potential for simple, rapid, low-cost, non-destructive, on-site, and real-time tracking of various analytes, with the advantages of being easy to miniaturize, visual detection results, and lacking the need of expensive equipment, complex pretreatment processes, and toxic fluorescence probes, etc. The variety of enzyme-mimic nanomaterial-based colorimetric sensors have been developed for the detection of heavy metal ions, such as Hg^2+^ [21], Cu^2+^ [22], Ag^+^ [23], and Fe^2+^ [24]. However, the detection limits of these sensors are restricted only to micromolar (µM) or nanomolar (nM) levels.

In this paper, a simple chemical reduction method was performed to generate uniform-sized PtNPs using citrate as the capping molecule. An ultrasensitive and selective colorimetric sensor for the rapid detection of Ag^+^ was developed with a detection limit down to the picomolar (pM) level based on the peroxidase-mimetic activity of PtNPs. The oxidation of TMB catalyzed by PtNPs could be inhibited by the reduced Ag^0^. As a result, the quantitative detection of Ag^+^ would be obtained by recording the UV absorption of oxidized TMB. To the best of our knowledge, the proposed sensor showed the highest sensitivity for Ag^+^ detection compared to recently-reported colorimetric sensors. The practical application of the colorimetric sensor for the detection of Ag^+^ in real water samples was also investigated and satisfactory results were obtained.

## 2. Materials and Methods

### 2.1. Materials and Instruments

3,3’,5,5’-tetramethylbenzidine dihydrochloride (TMB·2HCl) was purchased from Beyotime Biotechnology Co., Ltd. (Shanghai, China). All the other chemicals, such as chloroplatinic acid (H_2_PtCl_6_), NaBH_4_, citric acid, AgNO_3_, and ethylene diamine tetraacetic acid (EDTA) etc., were purchased from Sinopharm Chemical Reagent Co., Ltd. (Beijing, China). The reagents were of analytical grade and used as received without further purification. The solutions were prepared using ultrapure water purified by Milli-Q biocel from Millipore China Ltd. (Shanghai, China).

The UV–VIS absorption spectra and kinetic studies were performed on a UV-2450 UV-VIS spectrometer (Shimadzu, Tokyo, Japan). Terephthalic acid (TA) assay was carried out by an F-4600 fluorescence spectrofluorometer (Hitachi, Tokyo, Japan). Transmission electron microscopy (TEM) images were characterized by a high-resolution transmission electron microscopy (HRTEM) on a Philips Tecnai G2 F20 microscope (Philips, Amsterdam, The Netherlands) with an accelerating voltage of 200 kV. Before measurement, samples were prepared by dropping the PtNPs suspension on the surface of carbon-coated copper grid and drying it in air. X-ray photoelectron spectra (XPS) characterization was measured by the ESCALAB 250Xi X-ray photoelectron spectroscopy (Thermo Fisher Scientific, Waltham, MA, USA) using monochromatic Al Ka radiation (hv = 1486.6 eV). X-ray diffraction (XRD) characterization was performed by a Rigaku X-ray diffractometer (D/Max-3C, Tokyo, Japan).

### 2.2. Synthesis of Citric Acid-Modified PtNPs

Typically, 1 mL of chloroplatinic acid (16 mM), 1 mL of sodium citrate (40 mM) and 38 mL deionized water were added into a 50 mL beaker and stirring for 30 min at room temperature. After that, 200 µL of NaBH_4_ (50 mM) was introduced to the mixture drop by drop. The solution changed from colorless to brownish-yellow during the reaction process. Finally, citric acid-modified PtNPs were obtained after continuous stirring at room temperature for 1 h.

### 2.3. Colorimetric Detection of Ag^+^

Briefly, 8 μL of PtNPs (1.25 mg/L), 40 μL different concentrations of Ag^+^, and an appropriate amount of deionized water were added into a 0.6 mL centrifuge tube. After reacting for 2 min, 200 μL of TMB (1.6 mM) and 100 μL of H_2_O_2_ (2 M) were added into the solution to initiate the chromogenic reaction. The total reaction volume was 400 μL and the reaction was continued for a further 10 min. Finally, the absorption spectra of the resulting solutions were recorded in the range from 500–800 nm, the highest absorption at 652 nm was used as detection signal. The specific experiment was performed as above, except that other ions were used instead of Ag^+^.

## 3. Results and Discussion

### 3.1. Sensing Principle of the Ag^+^ Colorimetric Sensor

**Scheme 1:**
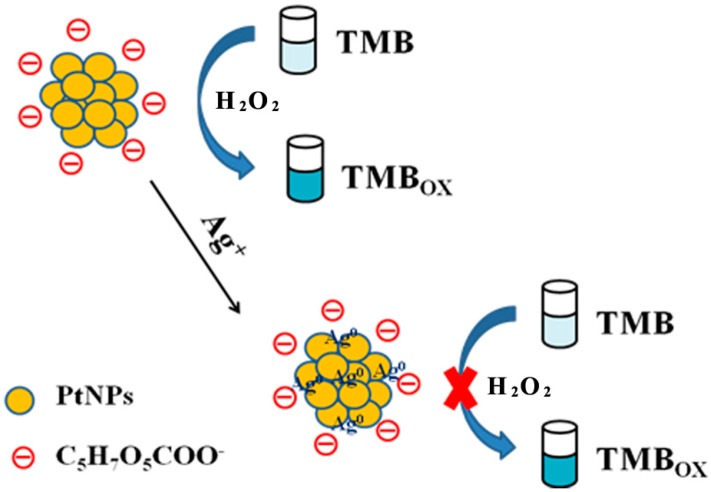
Schematic of the Ag^+^ colorimetric sensor based on citrate-modified PtNPs.

The schematic diagram of the Ag^+^ colorimetric sensor was shown in Scheme 1. By the use of citric acid as a stabilizer, chloroplatinic acid can be reduced by NaBH_4_ to generate stable and uniform PtNPs. The obtained PtNPs exhibit excellent peroxidase-mimic activity, which can catalyze the oxidization of TMB by H_2_O_2_ to produce a colored product. After addition of Ag^+^, the introduced Ag^+^ would be reduced by the capped citrate and deposited on the surface of PtNPs, which led to significant inhibition of the peroxidase-like activity of PtNPs. The specific Ag-Pt interaction provides the excellent selectivity toward Ag^+^ over other ions. Through measuring the maximum absorption of the oxidized TMB at 652 nm, an ultrasensitive, facile, and rapid colorimetric Ag^+^ sensor was established.

### 3.2. Characterization of the Formed PtNPs

**Figure 1:**
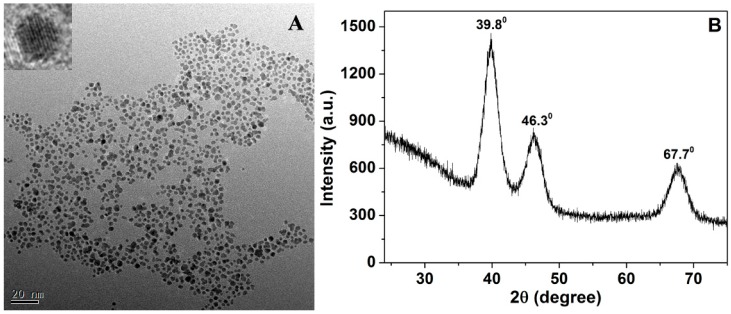
(**A**) TEM image of citric acid-modified PtNPs (Inset: HRTEM image of citric acid-modified PtNPs); and (**B**) XRD patterns of the PtNPs.

Transmission electron microscopy was used to investigate the morphological characteristics of the synthesized citric acid-modified PtNPs. As shown in Figure 1A, uniformly-dispersed PtNPs with narrow size distribution (diameter~2.5 nm) are observed. From the HRTEM image (inset Figure 1A), obvious lattice fringes of PtNPs can be noticed, which proves that the synthesized PtNPs have good crystal form. The measured lattice spacing is 0.223 nm, corresponding to the (1 1 1) facet of the Pt crystal [25]. The XRD patterns of the PtNPs are shown in Figure 1B. The diffraction peaks at angles of 39.8°, 46.3°, and 67.7° can be assigned to the (1 1 1), (2 0 0), and (2 2 0) facets of the face-centered cubic structures of platinum crystals ((JCPDS No. 4-802)) [26].

**Figure 2:**
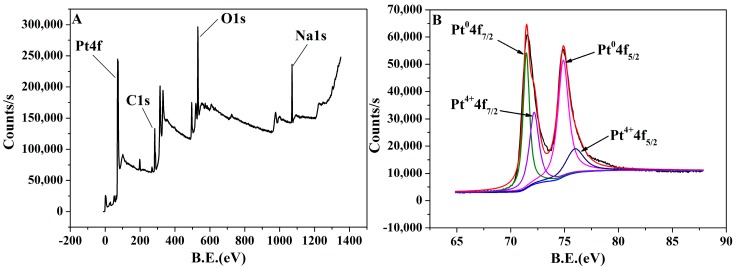
(**A**) The whole XPS spectrum of citrate-modified PtNPs; and (**B**) XPS spectrum showing the binding energy of Pt 4f.

XPS spectra were further performed to characterize the citric acid-modified PtNPs. Figure 2A shows the whole XPS spectrum of citrate-capped PtNPs. It can be seen that the elements of C, O, Na and Pt existed, indicating that the citric acid has been successfully modified on the surface of PtNPs. The binding energy of Pt 4f was shown in Figure 2B, the Pt 4f_7/2_ peak can be divided into two peaks with binding energy of 71.44 eV and 72.16 eV, corresponding to Pt^0^ and Pt^4+^, respectively. The Pt 4f_5/2_ peak also can be divided into two peaks at the binding energies of 74.88 eV and 75.97 eV, corresponding to Pt^0^ and Pt^4+^, respectively [27]. The ratio of Pt^0^ (59.7%) and Pt^4+^ (40.3%) on the PtNPs surface is determined as 1.48.

### 3.3. Catalytic Activity of PtNPs for TMB Oxidation

**Figure 3:**
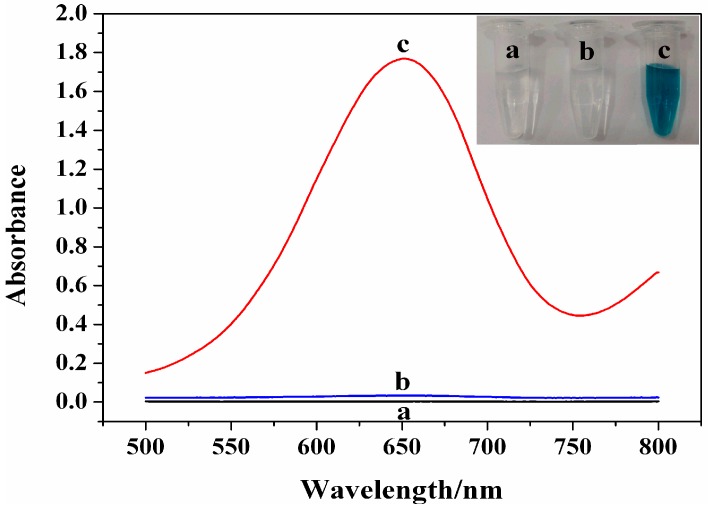
UV-VIS absorption spectrum of (a) TMB + H_2_O_2_; (b) TMB + PtNPs and (c) TMB + H_2_O_2_ + PtNPs (inset: the corresponding photographs).

**Figure 4:**
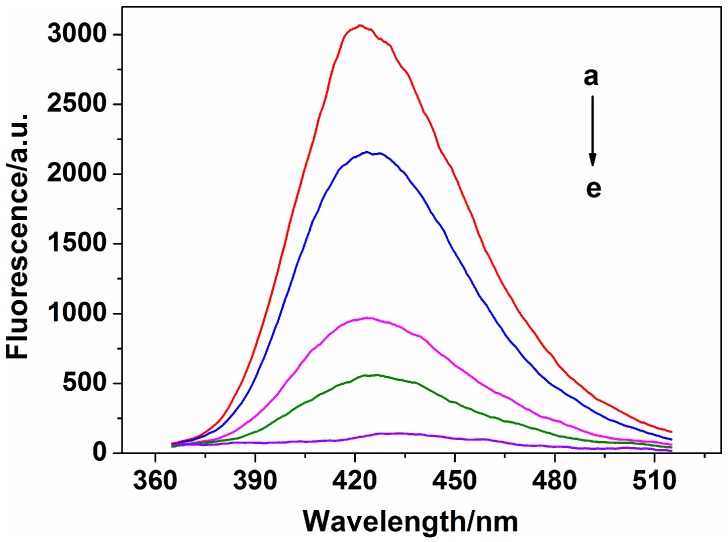
The effect of PtNPs on the formation of hydroxyl radicals in the H_2_O_2_/TA system. Samples were a mixture of 0.25 mM TA, 10 mM H_2_O_2_, and various concentrations of PtNPs (a) 0, (b) 5, (c) 12.5, (d) 25 and (e) 125 µg/L.

A series of control experiments were conducted to investigate the catalytic ability of citrate-modified PtNPs for the oxidation of TMB. As shown in Figure 3, the absorption signal was very small in the presence of TMB and H_2_O_2_ (curve a) or TMB and PtNPs (curve b) only, and the color of the solution is almost colorless (inset a, b). On the contrary, the absorption signal was remarkably increased when PtNPs, TMB, and H_2_O_2_ were coexistent in the solution (curve c), the color of the solution became deep blue (inset c). These experimental results showed that citrate-modified PtNPs have good peroxidase-mimetic properties to catalyze the oxidation of TMB by H_2_O_2_ effectively. Terephthalic acid (TA) was used to evaluate the effects of PtNPs on ·OH signal intensity, in which the added TA will react with ·OH to form a highly-fluorescent product, 2-hydroxyterephthalic acid (TAOH) [28]. As shown in Figure 4, a gradual decrease of the fluorescence intensity was observed while increasing the concentration of PtNPs, suggesting that the PtNPs reduced the ·OH radical signal, which is similar to the behavior of reported Co_3_O_4_ NPs [29], C-Dots [30] and MnO_2_ NPs [31]. In addition, experiments showed that the addition of high concentration of PtNPs into the mixture of TA and H_2_O_2_ resulted in a lot of bubbles (data not shown), indicating the PtNPs accelerated the decomposition of H_2_O_2_ to produce oxygen. These results and TA assays prove that PtNPs behave analogously to enzymes [32].

### 3.4. Inhibitory Effect of Ag^+^ on Catalytic Activity

**Figure 5:**
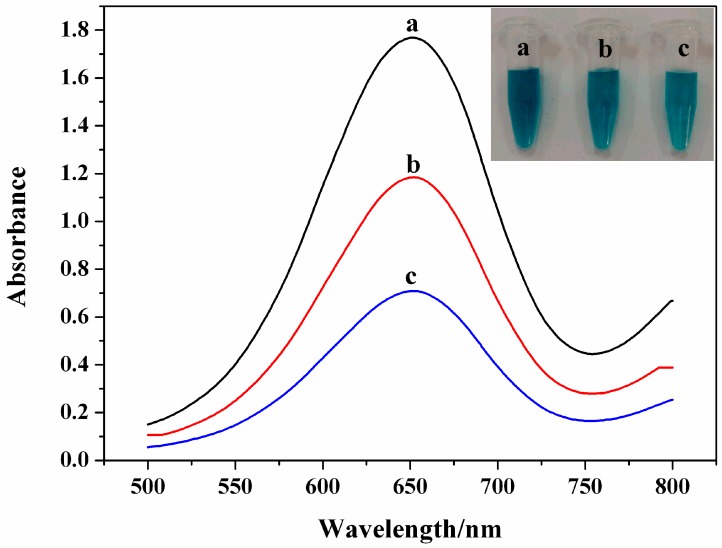
UV-VIS absorption spectrum of TMB at different concentrations of Ag^+^ (a) 0; (b) 1.5 nM; and (c) 3.0 nM.

**Figure 6:**
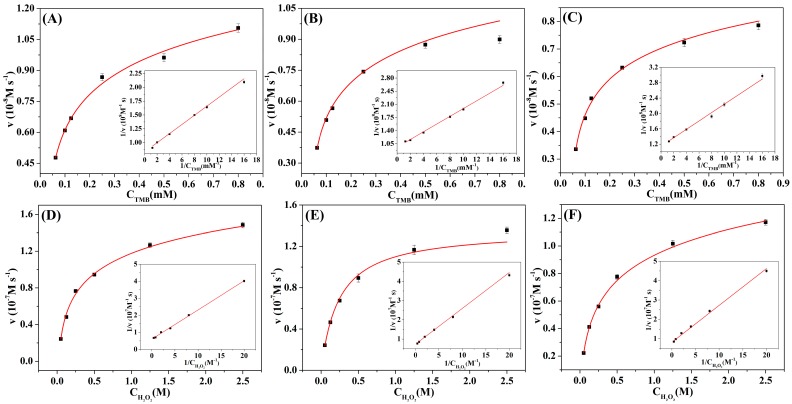
Steady-state kinetic analyses using the Michaelis–Menten model and Lineweaver–Burk model (insets) for PtNPs in the absence (**A**,**D**) and presence of 0.5 nM (**B**,**E**) and 2.0 nM (**C**,**F**) Ag^+^.

In order to examine the feasibility of the designed colorimetric sensor for the detection of Ag^+^, the absorption signals before and after addition of Ag^+^ were investigated. From Figure 5, it can be observed that a dark blue color solution was produced (inset a) with a strong absorption signal in the absence of Ag^+^ (curve a). After addition of 1.5 nM Ag^+^, the absorption signal was significantly decreased (curve b). At the same time, the color of the solution became lighter (inset b). When the Ag^+^ concentration was increased to 3.0 nM, the absorption signal was further inhibited (curve c), along with the color of the solution becoming shallower (inset c). These results indicated that the catalytic activities of PtNPs can be effectively inhibited by trace amounts of Ag^+^. Thus, a simple colorimetric sensor can be established for Ag^+^ detection with high sensitivity. The peroxidase-like activity of the PtNPs in the absence and presence of Ag^+^ was further investigated using steady-state kinetics (Figure 6). The apparent kinetic parameters were calculated based on the Michaelis-Menten equation: *v* = *V*_max_ × [S]/(*K*_m_ + [S]), where *ν* is the initial velocity, *V*_max_ is the maximal reaction velocity, [S] is the concentration of the substrate, and *K*_m_ is the Michaelis constant. *K*_m_ is an important parameter to evaluate the enzyme affinity to substrate. As shown in Table 1, the *K*_m_ value of the PtNPs increased, while the *V*_max_ value decreased after interaction with Ag^+^. These results indicated that Ag^+^-treated PtNPs have lower affinity to the substrates and weaker catalytic activity.

**Figure 7:**
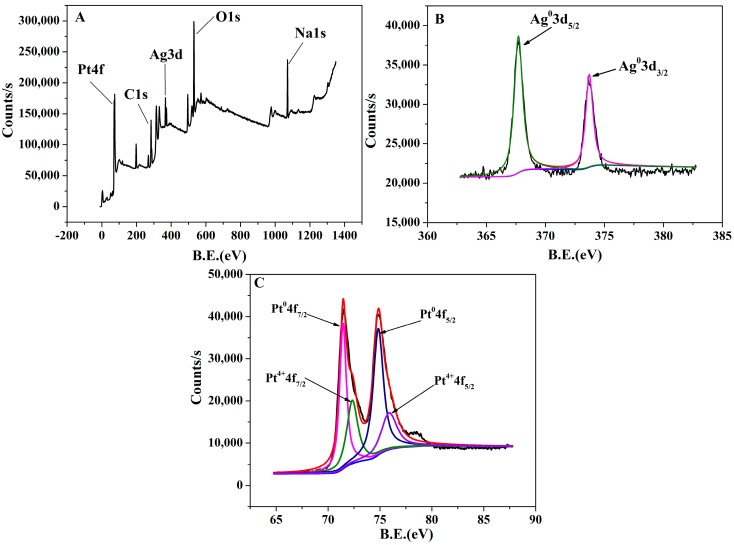
(**A**) The whole XPS spectrum; (**B**) Ag (3d) and (**C**) Pt (4f) XPS spectra of citrate-modified PtNPs after being treated with Ag^+^.

Similar with the interaction between Ag^+^ and gold nanoclusters, the possible mechanism of Ag^+^ to inhibit the catalytic activity of PtNPs could be related to a Pt-Ag metallic bond [33,34]. The added Ag^+^ can first interact with Pt to form a metallic bond, then be reduced by the modified citrate and deposited on the surface of PtNPs. We have performed an XPS spectrum of the citrate-modified PtNPs after being treated with Ag^+^ to investigate the inhibition mechanism. After interaction with Ag^+^, a new peak of Ag 3d could be observed in the XPS spectrum of PtNPs (Figure 7A). In addition, two well-characterized peaks appeared in the Ag 3d electron spectra of PtNPs after being treated with Ag^+^ (Figure 7B). The two signals of Ag 3d_5/2_ and Ag 3d_3/2_ that arose at binding energies of 367.70 and 373.72 eV corresponded to Ag^0^ [35,36]. Theoretically, Pt^0^ cannot be oxidized by Ag^+^ under conventional conditions due to the inert noble metal properties. The XPS spectra (Figure 7C) also indicated that addition of Ag^+^ do not have great effect on the ratio of Pt^0^ (58.7%) and Pt^4+^ (41.3%) on the PtNPs surface. Citrate is a thermal reduction reagent (reduction at near-boiling temperature), and the reduction of Ag^+^ with citrate is difficult to proceed at room temperature due to the weak reducibility [37,38]. Surprisingly, the citrate adsorbed on the surface of PtNPs could trigger Ag^+^ reduction catalyzed by the very reactive Pt surface atoms under mild conditions, which is similar to previous studies that showed the reduction of Hg^2+^ can be catalyzed by citrate-coated gold nanoparticles [39]. These results confirmed that the introduced Ag^+^ has been reduced to metallic Ag^0^ by the modified citrate, thereby causing changes in the surface chemistry of PtNPs and inhibiting the catalytic activity.

### 3.5. Optimization of Experimental Conditions

**Figure 8:**
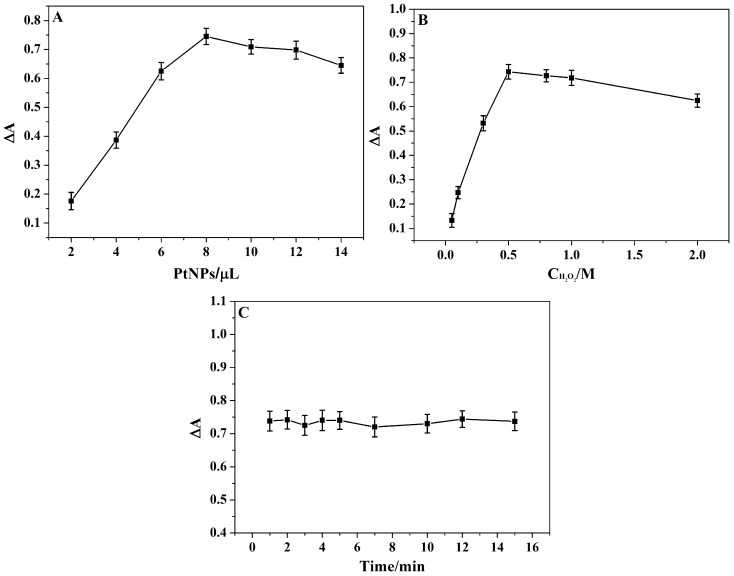
The effect of experimental conditions on the sensing performance (**A**) the volume of citrate-modified PtNPs; (**B**) the concentration of H_2_O_2_; and (**C**) the reaction time between Ag^+^ and PtNPs.

In order to obtain the best sensing performance, some experimental conditions were optimized. The absorption difference between A_0_ and A (recorded as ∆A) was used to evaluate the sensing performance, where A_0_ and A represent the absorption signal without and with the addition of 2.0 nM Ag^+^, respectively. We found that the amount of PtNPs has a great effect on the absorption signal (Figure 8A). The ∆A value was increased with the increase of PtNP volume up to 8 µL. However, with a further increase in the volume of PtNPs, the ∆A value started to decrease. According to these results, 8 µL PtNPs was used in subsequent experiments. The effect of H_2_O_2_ concentration on the developed sensor has also been investigated. H_2_O_2_, which acted as an oxidant, has played an important role in the oxidation of TMB. The ∆A value was remarkably increased with increasing H_2_O_2_ concentration from 0.05 to 0.5 M, then tends to decrease when the concentration of H_2_O_2_exceeds 0.5 M (Figure 8B). Therefore, 0.5 M H_2_O_2_ was used during the sensing process. The reaction time between Ag^+^ and PtNPs was also investigated. As shown in Figure 8C, the effect of Ag^+^ reaction time on the ∆A value is very small, which reflects that the interaction between Ag^+^ and citrate-modified PtNPs is fast. Taking into account the efficiency of the detection and ease of operation, we chose 2 min as the reaction time of Ag^+^.

### 3.6. Sensitivity of the Ag^+^ Sensing System

**Figure 9:**
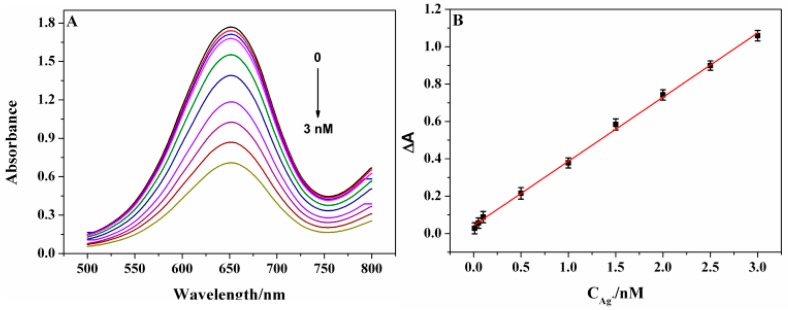
(**A**) Absorbance curves of the sensor for Ag^+^ at various concentrations; and (**B**) the corresponding calibration plot of absorbance values against the Ag^+^ concentrations (the error bars represent the standard deviation of three measurements).

To evaluate the sensitivity and dynamic range of the proposed colorimetric sensor for Ag^+^ detection, various concentrations of Ag^+^ were tested under the optimal conditions. As shown in Figure 9A, the absorption signal at 652 nm decreased with increasing concentration of Ag^+^. The more Ag^+^ that was added, the more Ag^0^ was formed, which greatly inhibits the catalytic activity of citrate-modified PtNPs. We obtained a good linear response of the absorption signal against the concentrations of Ag^+^ in the ranges from 0.01 to 3.0 nM with a correlation coefficient of 0.997 (Figure 9B). According to triplicate standard deviation over the blank response (3σ), the detection limit of (LOD) Ag^+^ was estimated to be 7.8 pM, which was sensitive enough for Ag^+^ detection in drinking water.

The analytical performance of the present sensor was compared with other Ag^+^ detection methods. As shown in Table 2, the sensitivity of the proposed sensor was higher than that of recently-reported colorimetric, fluorescent and electrochemical methods. Such high sensitivity was attributed to the highly inhibitory effect of Ag^+^ on the catalytic activity of PtNPs. The proposed sensor is simple, rapid and economical due to the mild synthesis of PtNPs without the need of special reagents, such as nucleic acid and fluorochrome. The whole sensing process can be finished within twelve minutes.

### 3.7. Selectivity and Recovery Performance

**Figure 10:**
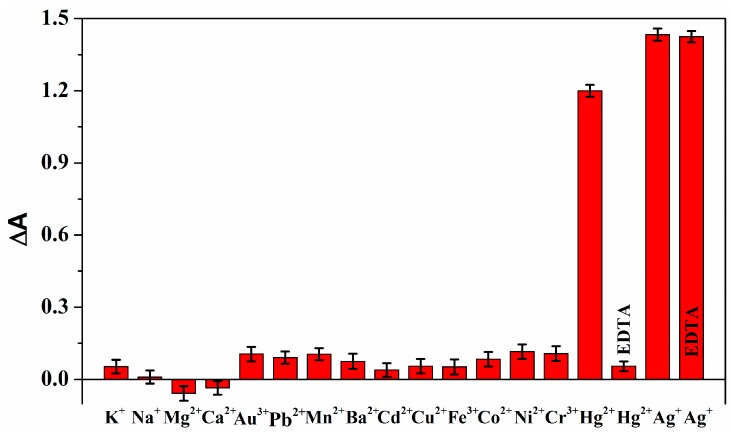
Selectivity investigation of the proposed sensor for Ag^+^ detection (the concentration of K^+^, Na^+^, Mg^2+^, Ca^2+^ : 0.5 mM, Ag^+^ and Hg^2+^: 10 nM, other ions: 1.0 µM).

In order to investigate the selectivity of the proposed sensor, an interference study was performed with other metal ions that exist in the environment. The absorption intensity was tested under the same conditions, except that other metal ions were used instead of 10 nM Ag^+^. As shown in Figure 10, the absorption signal was greatly inhibited by Ag^+^. No significant decrease of the absorption signal was observed in the presence of above 100-fold concentration of other ions, except Hg^2+^. Due to the similar ionic radius and reduction potential between Hg^2+^ and Ag^+^, Hg^2+^ could be adsorbed on citrate-capped PtNPs and be reduced by the modified citrate [48,49]. Thus, Hg^2+^ would interfere with the detection. For Ag^+^ sensing, EDTA was chosen as a masking agent because it could form more stable complexes with Hg^2+^ than that with Ag^+^. After interaction with EDTA, the influence of Hg^2+^ was effectively eliminated. The absorption intensity in the presence of Ag^+^ is completely irreversible after the introduction of an excess concentration of EDTA, indicating that Ag^+^ interacts with PtNPs through stronger interaction forming an Ag-Pt metallic bond, similar to the Ag-Au metallic bond [33,50]. Therefore, specific detection of Ag^+^ can be accomplished by the citrate-modified PtNP-based assay. More importantly, the results of selective experiments are visible to the naked eye, thus no special instruments are required to distinguish the presence or absence of Ag^+^.

The practical application of the designed colorimetric sensor was also tested through determination of Ag^+^ in river water samples by the standard addition method. The collected Minjiang River water samples were filtered with a 0.22 µm membrane to remove insoluble matter before Ag^+^ detection. Spiked samples were prepared with the further addition of different concentrations of standard Ag^+^ to the river water. Each Ag^+^ spiked sample was repetitively measured three times. The results are shown in Table 3. The recovery values ranging from 98.0% to 105.0% were obtained, and the relative standard deviation (RSD) was lower than 7%. These results revealed that the developed sensor has acceptable accuracy and reproducibility for the sensing of Ag^+^ in real samples.

## 4. Conclusions

In summary, a facile and simple colorimetric sensor was successfully developed for the ultrasensitive detection of Ag^+^ with a detection limit down to the pM level. Through efficient and specific inhibition of the peroxidase-mimic activity of citrate-modified PtNPs, highly sensitive and selective detection of Ag^+^ in real water samples can be achieved. The whole test can be completed within twelve minutes. There is no need of any expensive regents, complicated separation, or labeling processes during the sensing procedure. Thus, the fabricated sensor is rapid and economical. More importantly, through analysis of Ag^+^, the fabricated sensor can provide a new general, high-throughput, and portable sensing platform for indirect detection of various analytes.

## Figures and Tables

**Table 1:** Comparison of the kinetic parameter of PtNPs before and after being treated with Ag^+^.

Ag^+^ (nM)	TMB (*K*_m_/mM)	TMB (*V*_max_/M S^−1^)	H_2_O_2_ (*K*_m_/mM)	H_2_O_2_ (*V*_max_/M S^−1^)
0	0.0995	1.201 × 10^−^^8^	230.8	1.656 × 10^−^^7^
0.5	0.1077	1.045 × 10^−^^8^	255.9	1.372 × 10^−^^7^
2.0	0.1652	0.872 × 10^−^^8^	283.6	1.215 × 10^−^^7^

**Table 2:** Comparison of our present work with other methods for Ag^+^ detection.

Methods	Probes	Linear Range	LOD	References
Colorimetric	Peptide-AuNPs	10~1000 nM	7.4 nM	[40]
Colorimetric	Au@PtNPs	5~100 nM	2.0 nM	[41]
Colorimetric	DNA-AuNPs	1~1000 nM	0.24 nM	[42]
Colorimetric	AuNPs	1~9 μM	0.41 μM	[43]
Colorimetric	BSA-Au clusters	0.5~10 μM	204 nM	[23]
Fluorescence	Proflavine-DNA/MnO_2_	30~240 nM	9.1 nM	[44]
Fluorescence	DSAI/C-rich DNA	0~4.0 μM	155 nM	[45]
Electrochemistry	DNA/AuNPs	0.1~40 nM	0.05 nM	[46]
Electrochemistry	DNA/Fe_3_O_4_-AuNPs	10~150 nM	3.4 nM	[47]
Colorimetric	Citrate-modified PtNPs	0.01~3.0 nM	7.8 pM	This work

**Table 3:** The analysis of Ag^+^ in the real water samples.

Sample	Add (nM)	Found (nM)	Recovery (%)	RSD (%)
River water	0.20	0.21	105.0	6.7
	1.00	1.03	103.0	5.4
	2.00	1.96	98.0	5.1
